# Supplementary material for: QT prolongation risk factors and a monitoring strategy in rifampicin-resistant tuberculosis: Findings from the STREAM Stage 2 trial
Source: PLOS Glob Public Health. 2026 Feb 10;6(2):e0005113. doi: 10.1371/journal.pgph.0005113 (PMC12890085; doi:10.1371/journal.pgph.0005113)
Supplement: S1 Text — (DOCX) [file pgph.0005113.s001.docx]

**Supplementary appendix**

Table A in S1 Text. TSH change from baseline to week 40 between prolongation groups

| QT or QTcF ≥500ms |  |
| --- | --- |
| Event (n=24) | Mean difference from baseline (SD): 0.37 (1.857) |
| No Event (n=431) | Mean difference from baseline (SD): 0.36 (1.296) |
|  | Two-sided t test p-value: 0.979 |

Table B in S1 Text. Electrolyte abnormality by prolongation group

|  | No prolongation | Prolongation |
| --- | --- | --- |
| Electrolyte abnormality (Grade ≥1) at baseline | 66 (13%) | 1 (4%) |
| No electrolyte abnormality (Grade ≥1) at baseline | 462 (87%) | 27 (96%) |
| Electrolyte abnormality (Grade ≥1) on treatment | 344 (65%) | 16 (57%) |
| No electrolyte abnormality on treatment | 184 (35%) | 12 (43%) |
|  |  |  |
| TOTAL | 528 | 28 |

Figure A in S1 Text. Relationship between timing of electrolyte abnormality and QT event

Table C in S1 Text. Performance of the STREAM Stage 1 ECG monitoring strategy in the six-month regimen for STREAM Stage 2

|  | No QT prolongation, N (%) | QT prolongation, N (%) | Overall, N (%) | P-value |
| --- | --- | --- | --- | --- |
| 4-hour ECG reading | | | | |
| QTcF <425ms | 116 (83) | 1 (25) | 117 (82) | 0.019 |
| QTcF ≥425ms | 38 (17) | 3 (75) | 26 (18) |  |
| Week 3 ECG reading | | | | |
| QTcF <430ms | 97 (84) | 1 (100) | 98 (84) | 1.000 |
| QTcF ≥430ms | 19 (16) | - | 19 (16) |  |

**Machine readings**

Table D in S1 Text. Performance of the STREAM Stage 1 ECG monitoring strategy for machine readings in the control regimen for STREAM Stage 2

|  | No QT prolongation, N (%) | QT prolongation, N (%) | Overall, N (%) | P-value |
| --- | --- | --- | --- | --- |
| 4-hour ECG reading | | | | |
| QTcF <425ms | 57 (71) | 2 (20) | 59 (66) | 0.003 |
| QTcF ≥425ms | 23 (29) | 8 (80) | 31 (34) |  |
| Week 3 ECG reading | | | | |
| QTcF <430ms | 51 (81) | 2 (100) | 53 (82) | 1.000 |
| QTcF ≥430ms | 12 (19) | - | 12 (19) |  |

Table E in S1 Text. Performance of the STREAM Stage 1 ECG monitoring strategy for machine readings in the oral regimen for STREAM Stage 2

|  | No QT prolongation, N (%) | QT prolongation, N (%) | Overall, N (%) | P-value |
| --- | --- | --- | --- | --- |
| 4-hour ECG reading | | | | |
| QTcF <425ms | 72 (79) | 5 (71) | 77 (79) | 0.640 |
| QTcF ≥425ms | 19 (21) | 2 (29) | 21 (21) |  |
| Week 3 ECG reading | | | | |
| QTcF <430ms | 48 (67) | 1 (20) | 49 (64) | 0.056 |
| QTcF ≥430ms | 24 (33) | 4 (80) | 28 (36) |  |

Table F in S1 Text. Performance of the STREAM Stage 1 ECG monitoring strategy for machine readings in the six-month regimen for STREAM Stage 2

|  | No QT prolongation, N (%) | QT prolongation, N (%) | Overall, N (%) | P-value |
| --- | --- | --- | --- | --- |
| 4-hour ECG reading | | | | |
| QTcF <425ms | 23 (77) | - | 23 (74) | 0.258 |
| QTcF ≥425ms | 7 (23) | 1 (100) | 8 (26) |  |
| Week 3 ECG reading* | | | | |
| QTcF <430ms | 21 (91) | - | 21 (91) | - |
| QTcF ≥430ms | 2 (9) | - | 2 (9) |  |
